# Metabolite accumulation and metabolic network in developing roots of *Rehmannia glutinosa reveals* its root developmental mechanism and quality

**DOI:** 10.1038/s41598-018-32447-6

**Published:** 2018-09-20

**Authors:** Yanqing Zhou, Ke Yang, Dandan Zhang, Hongying Duan, Yongkang Liu, Mengmeng Guo

**Affiliations:** 1 College of Life Sciences, Henan Normal University, Xinxiang, 453007 HN P. R. China; 2 Wen County Institute of Agricultural Sciences, Wenxian, 454881 HN P. R. China

## Abstract

*Rehmannia glutinosa* root contains many compounds with important medicinal properties and nutritional benefits, but only more than 140 compounds have been reported so far. Many other compounds and their accumulation and metabolic networks during its development remain unclear. In order to clarify them, its metabolic profiles at three different developmental stages were analyzed using untargeted LC-MS analysis. Multivariate analysis revealed that 434 metabolites differently accumulated in its different stages, suggesting different change trends. The metabolites having the same trend share common metabolic pathways, the metabolites showing increasing contents during its development have medical and nutritional values, and some mature root-specific metabolites may be better candidates for its quality control; 434 metabolites were mapped onto 111 KEGG pathways including 62 enzymes, whose increasing and decreasing patterns were shown during its development. Some metabolites complicatedly interacted with some enzymes and the top-10 pathways enriched from 111 KEGG pathways in network analysis. These findings extended the dataset of its identified compounds, and revealed that its development and quality were associated with the accumulation of different metabolites. Our work will lay the foundation for the better understanding of its chemical constituents, quality and developmental mechanism.

## Introduction

*Rehmannia glutinosa* is a medicinally important perennial herb. Its root is rich in bioactive compounds, and thus, used to treat fever, nervous conditions, diabetes, and hypertension; to strengthen liver function; to enhance the hematopoietic function and immune defense; and serves as an ingredient in tonics in Traditional Chinese Medicine^1^.

These bioactive compounds have high economic values, in part because of their noted medicinal effects^2^, so *R. glutinosa* root’s chemistry has been studied widely for a long time. Previous phytochemical investigations on *Rehmannia* species have led to the isolation and identification of iridoid glycosides, ionone glycosides, phenethyl alcohol glycosides, and several other components^3^. Among the *Rehmannia* species, *R. glutinosa* and its compounds are by far the best studied to date. Moreover, its new compounds are increasingly isolated and identified^2,4–8^. Now, we known that *R. glutinosa* contains more than 140 individual compounds, such as monoterpenoids, phenethylalcohol glycosides, and triterpenes^9^. Among these compounds, catalpol and verbascoside are used as index components of *R. glutinosa* in the Pharmacopoeia of the People’s Republic of China. Verbascoside possesses pharmacological bioactivities for human health, such as antioxidant, anti-inflammatory, antineoplastic, wound-healing and neuroprotective properties^10^. Catalpol has important roles to play in the treatment of many diseases, including kidney diseases, neurodegenerative diseases and diabetes^11^. It is well-known that plant metabolomes are composed of over 200,000 metabolites that control plant development, and even *Arabidopsis* contains some 5000 metabolites, so *R. glutinosa* should contain more than these known compounds. In recent years, multiple ‘-Omics’approaches^12^ have been applied to *R. glutinosa* to better understand the formation and development of tuberous roots, to mine genes related to bioactive components, and to establish the biosynthesis pathways of bioactive components in its root^10,11,13,14^.

Metabolomics is an emerging field of “-Omics^12^” research specializing in the near global analysis of small molecular metabolites found in living organisms, and can simultaneously detect many endogenous metabolites, and thus provide a systematic description of the metabolic profile. Metabolomics has been successfully applied to investigate food and tea^15^, the processing chemistry of study on *Rehmanniae* Radix^1^, physiological mechanisms and biomarker discovery^16^. Untargeted analysis is the most commonly used strategy in liquid chromatography−mass spectrometry (LC-MS)-based metabolomics studies. With a high-resolution mass spectrometer, untargeted analysis can detect many metabolites in a non-prejudiced way and provide accurate mass estimates to facilitate compound identification^16^. For example, it was used to determine pesticide residues in globe artichoke^17^ and *Annonaceous acetogenins*^18^, to discriminate *Salvia miltiorrhiza* samples according to genotype^19^, and to identify the active metabolites in the hydroalcoholic extract of *With aniasomnifera* root^20^.

In this study, novel metabolic profiles of *R. glutinosa* roots at its three developmental stages were compared by untargeted LC-MS-based metabolomics approach coupled with multivariate analysis and univariate to analyze metabolite accumulation and metabolic network in developing roots of *Rehmannia glutinosa*.

## Results

### Sample collection and developmental stages

The elongating root (ER), expanding or thickening root (TR), and mature root (MR) were collected in turn from *R. glutinosa* cultivar “Jinjiu (03–2)” plants grown in farmed land in Wen County, Henan, China, at the elongation stage (E) on May20, the expanding or thickening stage (T) on August 20, and the mature stage (M) on November 10, 2016.

### Construction of the data matrix

In order to construct a data matrix, the peaks were extracted from the original data by XCMS software. The data matrix includes the sample name (ID) and the mass-to-charge ratio(m/z), retention time(r.t.), detection model, and peak intensity. This matrix contains a total of 3684 m/z features, including 2506 m/z features in the positive ionization mode, and 1178 m/z features in the negative ionization mode (Supplementary Table S1). Based on m/z features, catalpol (m/z = 361.1) and verbascoside or acteoside (m/z = 623) were reversely identified from this matrix. This matrix will be used for the following analyses.

### Multivariate analysis

**Figure 1:**
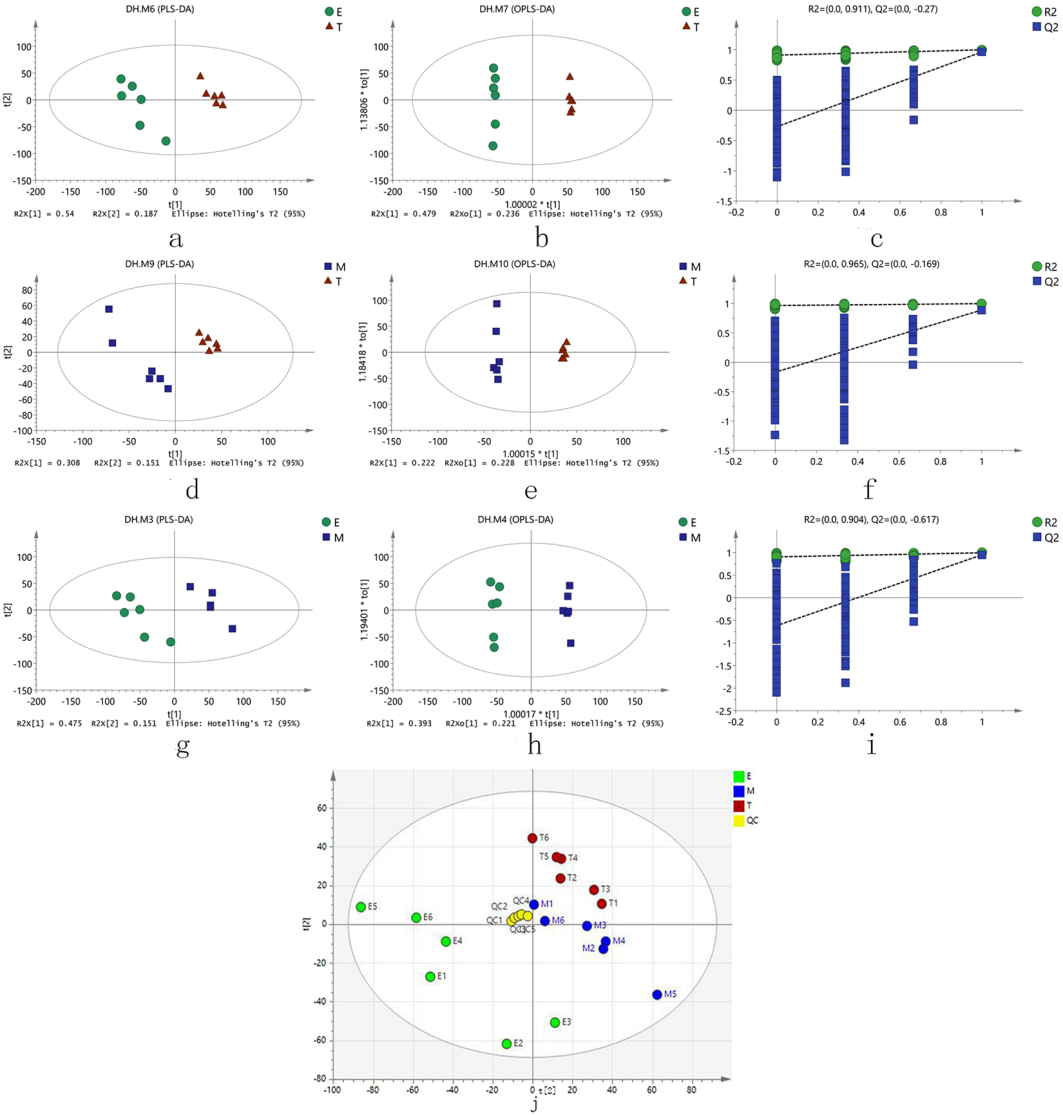
Score scatter plots of PCA, PLS-DA, OPLS-DA and validation plots of OPLS-DA for three comparative groups. (**a**) PLS-DA for TR/ER comparative group. (**b**) OPLS-DA for TR/ER comparative groupl. (**c**) validation plots of OPLS-DA for TR/ER comparative group. (**d**) PLS-DA for MR/TR comparative group. (**e**) OPLS-DA for MR/TR comparative group. (**f**) Validation plots of OPLS-DA for MR/TR comparative group. (**g**) PLS-DA for ER/MR comparative group. (**h**) OPLS-DA for ER/MR comparative group. (**i**) Validation plots of OPLS-DA for ER/MR comparative group. (**j**) Score scatter plots of PCA for TR/ER comparative group, MR/TR comparative group and ER/MR comparative group

In order to study the relations between multiple independent variables and multiple dependent variables, PCA, PLS-DA and OPLS-DA models are used to analyze the data in the data matrix. For the three comparative groups of ER/TR, ER/MR, and TR/MR, their score plots from the PCA, PLS-DA, and OPLS-DA, and the validation plots of OPLS-DA models were constructed (Fig. 1). All parameters for these models are summarized in Table 1. It is evident that all the values for Hotelling’s T2 are 95% (confidence interval, which stands for the degree of confidence, the bigger, the better), all for Q2 are < 0 (Q2 = −0.617 to −0.169. general requirement is Q2 < 0 in external validation, overfitting is avoided), all for R2X are >0.4 (general requirement is R2X > 0.4, the model is good.), and all for R2 are close to 1 (The closer to 1 R2 value, the better the model; general requirement is R2 > 0.5 in internal validation).

**Table 1:** The parameters for the assessment of these models.

NO. Model		Type	A	N	R2X(cum)	R2Y(cum)	Q2(cum)	R2	Q2
All	M1	PCA-X	4	23	0.689		0.399		
em	M2	PCA-X	2	12	0.605		0.344		
em	M3	PLS-DA	4	12	0.839	0.994	0.966		
em	M4	OPLS-DA	1 + 3 + 0	12	0.839	0.994	0.951	0.904	−0.617
et	M5	PCA-X	2	12	0.649		0.455		
et	M6	PLS-DA	3	12	0.79	0.998	0.988		
et	M7	OPLS-DA	1 + 3 + 0	12	0.822	0.999	0.979	0.911	−0.27
mt	M8	PCA-X	2	12	0.522		−0.00798		
mt	M9	PLS-DA	3	12	0.667	0.992	0.921		
mt	M10	OPLS-DA	1 + 3 + 0	12	0.758	0.998	0.889	0.965	−0.169

Note: A stands for the PC numbers while each model is constructed, N for the numbers of samples analyzed, em for the comparative group E/M, et for the comparative group E/T, mt for the comparative group M/T, M1-M10 for Model1–10, R2X(cum) for the interpretation rate of each model in the X axis direction in multivariate statistical analysis modeling, R2Y(cum) for the interpretation rate of each model in Y axis direction in multivariate statistical analysis modeling, Q2(cum) for the prediction rate of each model, R2 for the intercept value of the Y axis and the regression line, which is obtained when Linear regression analysis between the Y matrix of the original classification, the Y matrices of N times′ different permutations and R2Y was conducted during model validation, and Q2 for the intercept value of the Y axis and the regression line, which is obtained when Linearregression analysis between the Y matrix of the original classification, the Y matrices of N times′ different permutations and Q2Y was conducted during model validation; for Q2 in external validation, general requirement is that Q2 < 0, overfitting is avoided. For R2 in internal validation, general requirement is that R2 > 0.5, the closer to 1R2, the better the model. For R2X, general requirement is that R2X > 0.4, the model is good.

### Identified differential metabolites

To screen the differential metabolites between any two comparative root groups, we integrated the results of the multivariate and univariate to obtain the criteria for screening the differential metabolites between any two comparative root groups: VIP > 1 for the first principal component in the OPLS-DA, *P*-value < 1 and Fold change (FC) > 1. FC > 1 indicates that differential metabolite are up-regulated, whereas FC < 1 indicates that differential metabolite are down-regulated. Based on these criteria, the differential metabolites among three comparative groups are identified (SupplementaryTable S2). Among these differential metabolites, catalpol is a differential metabolite (ID = 124, m/z = 401.0824627, C15H22O10, VIP >1, up-regulated 3.45 times) in TR/ER and a differential metabolite (ID = 1185, m/z = 385.1, C15H22O10, VIP >1, up-regulated 1.85 times) in MR/TR, However, verbascoside or acteoside is not a differential metabolite (Supplementary Table S2). There were 562 differential metabolites including197 differential metabolites in the ER/MR comparative group, and 252 in ER/TR and 113 in TR/MR. Among these 562 differential metabolites, 369 were non-repetitive differential metabolites between any two comparative groups. After the differential metabolites with “/” were removed, 434 differential metabolites were identified. They include 149 in ER/MR, 200 in ER/TR, and 85in TR/MR. Among these 434, 294 were non-repetitive differential metabolites between any two comparative groups. Among 294 differential metabolites, 281 were common among three comparative groups (Supplementary Table S6). Therefore, several hundred differential metabolites over *R. glutinosa* root development were identified.

### KEGG pathway analysis

**Figure 2:**
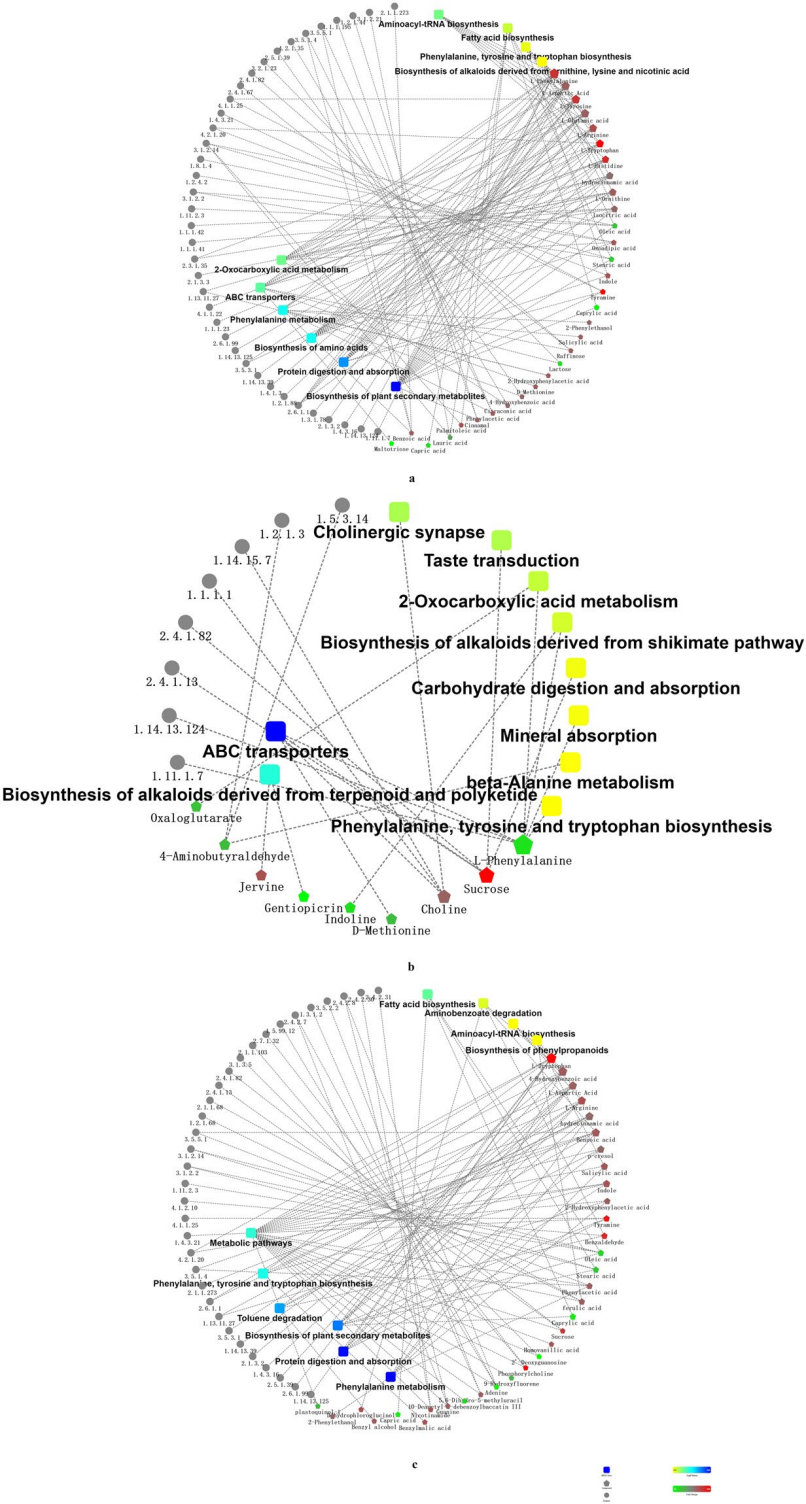
Network Analysis for TR/ER, TR/MR, MR/ER comparative groups. The network diagrams include boxes (the top-10pathways), dots (enzymes), rounded rectangles (compounds), and lines. The lines between these rounded rectangles and dots or boxes indicate their confirmed interactions (no lines are shown for unconfirmed interactions). The rounded rectangles represent the bioprocesses, cellular localization and molecular functions, or signaling pathways; lines between the dots and the rounded rectangles represent associations or participation; red represents an increasing quantity; green represents a decreasing quantity; and the yellow-to-blue gradient represents significance going from low to high. The numbers below the dots represent Enzyme Commission (EC) numbers. (**a**) Network Analysis for the TR/ER comparative group: benzoate O-methyltransferase (2.1.1.273), dodecanoyl-[acyl-carrier-protein] hydrolase (3.1.2.21), cinnamoyl-CoA reductas (1.2.1.44), cinnamyl-alcohol dehydrogenase (1.1.1.195), nitrilase (3.5.5.1), amidase (3.5.1.4), (R)-2-methylmalate dehydratase (4.2.1.35), 4-hydroxybenzoate polyprenyltransferase (2.5.1.39), beta-galactosidase (3.2.1.23), galactinol–sucrose galactosyltransferase (2.4.1.82), galactinol–raffinosegalactosyltransferase (2.4.1.67), Arginase (3.5.3.1), tyrosine decarboxylase (4.1.1.25), primary-amine oxidase (1.4.3.21), tryptophan synthase (4.2.1.20), oleoyl-[acyl-carrier-protein] hydrolase (3.1.2.14), dihydrolipoyl dehydrogenase (1.8.1.4), oxoglutarate dehydrogenase (succinyl-transferring) (1.2.4.2), palmitoyl-CoA hydrolase (3.1.2.2), plant seed peroxygenase (1.11.2.3), isocitrate dehydrogenase (NADP+) (1.1.1.42), isocitrate dehydrogenase (NAD+) (1.1.1.41), glutamate N-acetyltransferase (2.3.1.35), ornithine carbamoyltransferase (2.1.3.3), 4-hydroxyphenylpyruvate dioxygenase (1.13.11.27), Peroxidase (1.11.1.7) histidine decarboxylase (4.1.1.22), histidinol dehydrogenase (1.1.1.23), L-tryptophan–pyruvate aminotransferase (2.6.1.99), L-aspartate oxidase (1.4.3.16), tryptophan N-monooxygenase (1.14.13.125), nitric-oxide synthase (NADPH) (1.14.13.39), glutamate dehydrogenase [NAD(P)+] (1.4.1.3), L-glutamate gamma-semialdehyde dehydrogenase (1.2.1.88), aspartate transaminase (2.6.1.1), arogenate dehydrogenase (NADP+) (1.3.1.78), phenylalanine N-monooxygenase (1.14.14.40) and aspartate carbamoyltransferase (2.1.3.2). (**b**) Network Analysis for the TR/MR comparative group: polyamine oxidase (propane-1,3-diamine-forming) (1.5.3.14), aldehyde dehydrogenase (NAD+) (1.2.1.3), choline monooxygenase (1.14.15.7), alcohol dehydrogenase (1.1.1.1), galactinol–sucrose galactosyltransferase (2.4.1.82), sucrose synthase (2.4.1.13), anthraniloyl-CoA monooxygenase (1.14.13.124)and Peroxidase (1.11.1.7). (**c**) Network Analysis for the MR/ER comparative group: NAD + –protein-arginine ADP-ribosyltransferase (2.4.2.31), NAD + ADP-ribosyltransferase (2.4.2.30), hypoxanthine phosphoribosyltransferase (2.4.2.8), Dihydropyrimidinase (3.5.2.2), dihydropyrimidine dehydrogenase (NADP+) (1.3.1.2), adenine phosphoribosyltransferase (2.4.2.7), cytokinin dehydrogenase (1.5.99.12), choline kinase (2.7.1.32), phosphoethanolamine N-methyltransferase (2.1.1.103), 5′-nucleotidase (3.1.3.5), galactinol–sucrose galactosyltransferase (2.4.1.82), sucrose synthase (2.4.1.13), caffeate O-methyltransferase (2.1.1.68), coniferyl-aldehyde dehydrogenase (1.2.1.68), oleoyl-[acyl-carrier-protein] hydrolase (3.1.2.14), tyrosine decarboxylase (4.1.1.25), palmitoyl-CoA hydrolase (3.1.2.2), plant seed peroxygenase (1.11.2.3), (R)-mandelonitrilelyase (4.1.2.10), tryptophan synthase (4.2.1.20), Amidase (3.5.1.4), Arginase (3.5.3.1), primary-amine oxidase (1.4.3.21), 4-hydroxybenzoate polyprenyltransferase (2.5.1.39), benzoate O-methyltransferase (2.1.1.273), aspartate transaminase (2.6.1.1), 4-hydroxyphenylpyruvate dioxygenase (1.13.11.27), nitric-oxide synthase (NADPH) (1.14.13.39), aspartate carbamoyltransferase (2.1.3.2), Nitrilase (3.5.5.1), L-aspartate oxidase (1.4.3.16), L-tryptophan–pyruvate aminotransferase (2.6.1.99) and tryptophan N-monooxygenase (1.14.13.125).

**Figure 3:**
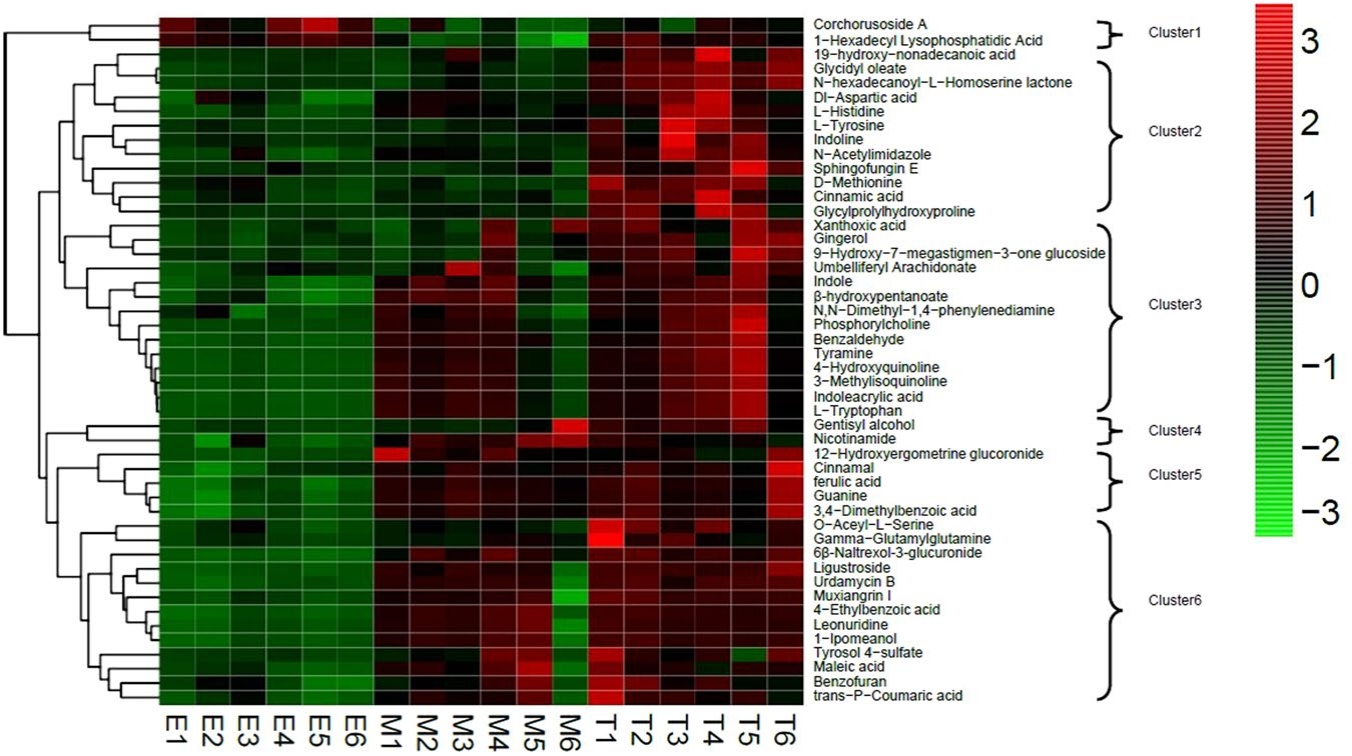
Heatmap of 48 identified differential metabolites. The heatmap is a false color image, with a dendrogram added to the left side. In the heatmap, rows represent differential metabolites and columns represent the samples. The dendritic structures to the left side represent the clustering relationships of similarities among the differential metabolites. T1 to T6, M1 to M6, and E1 to E6 represent six repeated experiments with TR, MR and ER, respectively.

434 differential metabolites were mapped with the KEGG database^21–24^ onto the KEGG pathways in which increased or decreased differential metabolites and enzymes are shown (Supplementary Tables S3–S5). They were identified by comparing the metabolites of roots between any two of three developmental stages of *R. glutinosa plants*, and then mapped onto 111 KEGG pathways, which included 62 enzymes (Table 2). The top 10 enriched KEGG pathways form each comparative group of *Rehmannia glutinosa* root were sorted by data and over-represented by system default operation so that we can narrow the scope of multiple KEGG pathways and focus on studying the top-10 enriched KEGG pathways, (1) In the ER/TR comparative group, the top-10 KEGG pathways include the biosynthesis of plant secondary metabolites, protein digestion and absorption, biosynthesis of amino acids, phenylalanine metabolism, ABC transporters, 2-oxocarboxylic acid metabolism, aminoacyl-tRNA biosynthesis, fatty acid biosynthesis, phenylalanine and tyrosine and tryptophan biosynthesis, and biosynthesis of alkaloids derived from terpenoid and polyketide, and had differences that were highly significant at the 0.01 level (i.e., *P* < 0.01) (Fig. 2); (2) In the TR/MR comparative group, the top-10 KEGG pathways include the ABC transporters, biosynthesis of alkaloids derived from terpenoid and polyketide, cholinergic synapse, taste transduction, 2-oxocarboxylic acid metabolism, biosynthesis of alkaloids derived from skikimate pathway, carbohydrate digestion and absorption, mineral absorption, beta-alanine metabolism and phenylalanine, and tyrosine and tryptophan biosynthesis. Among them the first two KEGG pathways were highly significant (*P* < 0.01), the last four were not significant, and the remaining four were significant (*P* < 0.05) (Fig. 1); (3) In the ER/MR comparative group, the top-10 KEGG pathways include the phenylalanine metabolism, protein digestion and absorption, biosynthesis of plant secondary metabolites, toluene degradation, phenylalanine and tyrosine and tryptophan biosynthesis, metabolic pathways, fatty acid biosynthesis, aminobenzoate degradation, aminoacyl-tRNA biosynthesis, and biosynthesis of phenylpropanoids, and have differences that were highly significant (*P* < 0.01) (Fig. 2).

**Table 2:** Functional analyses of identified differential metabolites.

Comparative groups	TN	NUM	NDM	NUMKFN	NDMKFN	NUMKPE/KP	NDMKPE/KP
TR/ER	200	111	89	56	43	24/37	11/21
MR/TR	85	28	57	3	19	1/1	4/12
ER/MR	149	66	83	35	33	16/23	6/17

TN, NUM, NDM, NUMKFN, NDMKFN, NUMKPE/KP and NDMKPE/KP stand for total number, number of increased metabolites, number of decreased metabolites, number of increased metabolites with known formulas and names, number of decreased metabolites with known formulas and names, number of increased metabolites with KEGG pathways and enzymes/with KEGG pathways, and number of decreased metabolites with KEGG pathways and enzymes//with KEGG pathways.

However, only one KEGG pathway, phenylalanine and tyrosine and tryptophan biosynthesis, was common among the three comparative groups. Four KEGG pathways, including phenylalanine, tyrosine and tryptophan biosynthesis, ABC transporters, 2-oxocarboxylic acid metabolism and biosynthesis of alkaloids derived from terpenoid and polyketide, were common in the TR/MR and ER/MR groups (Figs 1 and 2). Nevertheless, in the ER/TR and ER/MR groups, there were six common KEGG pathways: phenylalanine, tyrosine and tryptophan biosynthesis, phenylalanine metabolism, protein digestion and absorption, biosynthesis of plant secondary metabolites, fatty acid biosynthesis and aminoacyl-tRNA biosynthesis (Figs 1 and 2). Generally, several different KEGG pathways were shared between any two comparative groups. In a word, a total of KEGG pathways including enzymes among three comparative groups and the top-10 KEGG pathways in each comparative group were mined.

### Metabolic network analyses

Network analysis was conducted in order to reveal them olecular network and mechanism of the interaction of the top-10 KEGG pathways with related enzymes and compounds. Each network shows the interactions of compounds with enzymes or/and these pathways, and the FC changes and changes of log_2_ (*P*-value) features of these pathways (Fig. 2). For three comparative groups, the compounds interact with these pathways and/or enzymes, but no enzyme interacts with these pathways. There were three differences among the three comparative groups. First, for the TR/ER comparative group, the network includes 38 enzymes, 10 pathways, and 31compounds. One compound may interact with 1–8 pathways and/or enzymes; one pathway may interact with 2–17 compounds; and one enzyme may interact with 1–5 compounds. The FCs of compounds varied greatly. In the ABC transporters and biosynthesis of plant secondary metabolites, the levels of most compounds were increased, but the levels of lactose and maltotriose were decreased under the action of beta-galactosidase; the levels of oleic acid, stearic acid, and caprylic acid were all decreased under the action of fatty acyl-ACP thioesterase A. In fatty acid biosynthesis, the levels of capric acid, caprylic acid, lauric acid, and oleic acid were decreased under the action of both palmitoyl-protein thioesterase and fatty acyl-ACP thioesterase A. In the remaining eight KEGG pathways, the levels of metabolites were generally increased. Moreover, the changes in metabolite levels were the same as those in network analysis. For example, the levels of L-tryptophan and indole were increased under the action of tyrosine decarboxylase in the pathway of phenylalanine, tyrosine, and tryptophan biosynthesis, while the levels of phenylacetic acid, benzoic acid, 2-hydroxybenzoic acid, and D-methionine were increased under the action of 4-hydroxyphenylpyruvate dioxygenase in the phenylalanine metabolism pathway (Fig. 2). Second, for the MR/TR comparative group, the network includes eight enzymes, 10 pathways, and nine compounds. One compound may interact with 1–4 pathways and/or 0–3 enzymes; one pathway may interact with 1–2 compounds; and one enzyme may interact with one compound. The FCs of the compounds varies little. In the metabolic pathway, the levels of most compounds were increased, but the levels of 2-deoxyguanosine, 9-hydroxyfluorene, and plastoquinol were all decreased under the action of 5,6-dihydro-5-methyluracil 5′-nucleotidase, cytokinin dehydrogenase, and hypoxanthine phosphoribosyl transferase. In the biosynthesis of plant secondary metabolites, the levels of most compounds were increased, but the levels of oleic acid, stearic acid, and caprylic acid were decreased under the action of palmitoyl-protein thioesterase. In fatty acid biosynthesis, the levels of nicotinamide, oleic acid, stearic acid, and caprylic acid were decreased. In the remaining seven pathways, the levels of most compounds were increased under the action of both tyrosine decarboxylase and tryptophan synthase (Figs 1 and 2). Moreover, the changes in metabolite levels in the top-10 KEGG pathways were the same as those revealed by the network analysis. For example, the levels of L-tryptophan and indole were increased under the action of tyrosine decarboxylase in the pathway of phenylalanine, tyrosine, and tryptophan biosynthesis; the levels of phenylacetic acid, benzoic acid, 2-hydroxybenzoic acid, and D-methionine were increased under the action of 4-hydroxyphenylpyruvate dioxygenase in the phenylalanine metabolism pathway. In the biosynthesis of phenylpropanoids pathway, the levels of ferulic acid and salicylic acid were increased under the action of caffeic acid 3-O-methyltransferase. Third, for the ER/MR comparative group, the network includes 33 enzymes, 10 pathways, and 33 compounds. One compound may interact with 1–4 pathways and/or enzymes; one pathway may interact with 3–23 compounds; and one enzyme can interact with 1–2 compounds. The FCs of compounds varied widely. In the pathways of ABC transporters and biosynthesis of plant secondary metabolites, the levels of sucrose and choline were increased under the action of cinnamyl-alcohol dehydrogenase, raffinose synthase, and sucrose synthase. In the cholinergic synapse, choline level was increased. In beta-alanine metabolism, the levels of aldehyde dehydrogenase (NAD+), polyamine oxidase (propane-1,3-diamine-forming), and 4-Aminobutyraldehyde were decreased. In the biosynthesis of alkaloids derived from shikimate pathway, the level of jervine was increased whereas the level of gentiopicrin was decreased. In the pathways of taste transduction and carbohydrate digestion and absorption, the sucrose level was increased. In the pathways of 2-oxocarboxylic acid metabolism, biosynthesis of alkaloids derived from the shikimate pathway, mineral absorption, and phenylalanine, and the tyrosine and tryptophan biosynthesis, the L-phenylalanine level was decreased (Fig. 2). These results suggested that there should be complicated molecular networks and mechanisms of the interactions of the top 10 KEGG pathways with related enzymes and different metabolites.

### Heatmap of 281 common differential metabolites

In order to express the clustering relationships between differential metabolites, the peak areas of 281common differential metabolites were used to construct heatmap, of which 48 as representatives were shown in Fig. 3. In the heatmap, (1) some metabolites having the same trend share common pathways. For example, Cluster3 is a class of metabolites with a rising trend from ER (low) to MR (medium) to TR (high), mainly including alkaloids, indoles, abscisic acid, fatty acids, benzoic acid, etc. They belong to the following the top-10 pathways: Metabolic pathways (map01100), Toluene degradation (map00623), Phenylalanine, tyrosine and tryptophan biosynthesis (map00400), Biosynthesis of plant secondary metabolites (map01060), Aminobenzoate degradation (map00627), etc; Cluster4 is a class of metabolites with a rising trend from ER (low) to MR (medium) to TR (high), mainly including gentiols, nicotinamide, etc. They are the metabolites of the following the top-10 pathways: Metabolic pathways (map01100), etc; Cluster5 is a class of metabolites with a rising trend from ER (low) to MR (medium) to TR (high), mainly including glycosides, amino acids, benzoic acid, etc. They are the metabolites of the following the top-10 pathways: Metabolic pathways (map01100), etc; Cluster6 is a class of metabolites with a trend from ER (low) to MR (high) to TR (low), mainly including amino acids, glycosides, peptides, alkaloids, benzoic acid, carboxylic acids, etc. They are the metabolites of the following the top-10 pathways: Biosynthesis of secondary metabolites (map01110), Biosynthesis of type II polyketide products (map01057), etc. In addition, Cluster2 is a class of metabolites with a rising trend from ER (low) to MR (medium) to TR (high). These metabolites mainly include fatty acids, amino acids, esters, peptides, etc., and mainly exist in the following metabolic pathways other than the top-10 pathways: Indole alkaloid biosynthesis (map00901), Biosynthesis of alkaloids derived from shikimate pathway (map01063), etc. (2) some metabolites showing increasing contents over root development are likely to have medical and nutritional values. For example, L-tryptophan and nicotinamide in MR with up-regulated expression have very good medicinal and nutritional value. L-tryptophan is a nutritional supplement for humans and an antioxidant. Tryptophan is a precursor of the neurotransmitter serotonin, important for human body, and also a kind of essential amino acids in the body. Tryptophan can be used as a tranquilizer regulating nutritional rhythms and improving pregnant women′ sleep, and as nutritional supplements and special infantmilk powder, etc; Nicotinamide is clinically used to treat arrhythmias associated with coronary heart disease, viral myocarditis, rheumatic heart disease, and a few digitalis poisonings. It can also be used to control pellagra, stomatitis and glossitis. (3)some metabolites may be better candidates for quality control for *Rehmannia glutinosa* roots than catalpol and verbascoside. For example, in the MR, there are 3 specific metabolites, such ascinnamic acid, 1-hexadecyl lysophosphatidic acid and 9-hydroxy-7-megastigmen-3-oneglucoside. Similarity settlement relationships between differential metabolites and similarity settlement relationships between samples were shown in Heatmap.

## Discussion

*Rehmannia glutinosa* root development is crucial for its yield, quality, and medicinal value after harvest, so considerable attention has been paid to its study via different methods. In terms of the “omics” approaches, the transcriptomics, proteomics, the micRNA transcriptome, and degradome have been used to elucidate the mechanisms of initiation and determination in the formation of tuberous roots in *R. glutinosa*, and to identify light signaling, calcium signaling, and other key cellular processes initiating the expansion of its fibrous roots. In addition, previous studies have identified the miRNAs that controlled the transition of its fibrous roots towards the tuberous roots, and 6794 differentially expressed unigenes during root development^14^, and have elucidated the regulation mechanisms of tuberous roots formation^12^. The untargeted LC-MS-based metabolomics approachis are widely used now to investigate plant metabolites and their networks. For example, it can discriminate *Salvia miltiorrhiza* samples according to genotype^19^, to identify the active metabolites in the hydroalcoholic extracts of *With aniasomnifera* root^20^, and to detect the major metabolite changes during manufacturing process of tea^25^.

In the present study, untargeted LC-MS-based metabolomics approach, multivariate analysis and univariate analysis were used to identify the differential metabolites (Supplementary Table S2). 434 non-repetitive differential metabolites were identified between any two of three comparative groups such as ER/TR, TR/MR, and ER/MR (Table 2), of which 281 were common differential metabolites among three root comparative groups. In previous study, more than 140 individual compounds from *R. glutinosa* had were discovered^9^. However, in the present study, so many differential metabolites were identified by means of a untargeted LC-MS-based metabolomics approach, showing whose high efficiency and the diversity and richness of the metabolites in *R. glutinosa* roots. What is more, so many differential metabolites indicate that the quality of *R. glutinosa* root is different among different developmental stages.

Because catalpol and verbascoside are designated as two important index components of *R. glutinosa* as mentioned above, either or both are often used to evaluate the quality of *R*. *glutinosa* at harvest time (i.e. the late October to the early November). Furthermore, it was found that the catalpol content in *R. glutinosa* followed an upward trend during its root development, with its highest content in its mature tuberous roots at its mature stage (the late October to the early November)^26–29^, the best period for their harvest which coincides with its traditional harvest period^29^. Meanwhile, it was also found that the verbascoside content in *R*. *glutinosa* root was also the greatest during the late October to the early November, coinciding with its traditional harvest time^29^. Nevertheless, it was also reported that the verbascoside content was on a downward trend during *R*. *glutinosa* root development^30^. On the other hand, some researcher investigated the relations of the harvest period to the contents of more metabolites other than catalpol and verbascoside in *R. glutinosa*, and found that the contents of monosaccharides, oligosaccharides and amino acids in *R. glutinosa* were greatest during the late October to the early November, which coincides with its traditional harvest time^29^. In the current study, catalpol and verbascoside were identified. Verbascoside is not differential metabolite but catalpol is a differential metabolite in *R. glutinosa* Cultivar Jinjiu (03–2). Our result about catalpol content change trend is consistent with previous reports^26–29^, but our result about verbascoside content change trend is inconsistent with previous reports^29,30^, showing that verbascoside content change trend may be different among different *R. glutinosa* cultivars. In addition, we discovered the contents of many differential metabolites in *R. glutinosa* followed an upward trend during its development, with its highest content in its mature tuberous roots at its mature stage (Fig. 2), and discovered some specific differential metabolites such as cinnamic acid, 1-hexadecyl lysophosphatidic acid, 9-hydroxy-7-megastigmen-3-one glucoside, which may be better candidates for quality control for *Rehmannia glutinosa* roots compared with catalpol and verbascoside.

From the network analysis, it was seen that not only the numbers of metabolites interacting with the top-10 KEGG pathways but also the numbers of enzymes with increased levels or decreased levels were different among these three comparative root groups (Fig. 2). The total number of metabolites and enzymes were ranked as follows: ER/TR > ER/MR > TR/MR comparative group. This ranking was also applied to the degree of complexity of the interaction of metabolites with the KEGG pathways and enzymes. Collectively, our findings revealed that (1) the numbers, increased and decreased levels, the different levels of the top-10 pathways, and clustering of differential metabolites underwent similar change patterns; (2) the interactions of differential metabolites with the enzymes and the top-10 pathways were complex; and (3) the different levels of the top-10pathways coincided with the increased and decreased levels of differential metabolites. Taken together, these accumulation change patterns and interaction mechanisms control *R. glutinosa* root development.

## Conclusion

The metabolomes of root samples from different developmental stages of *R*. *glutinosa* plants were analyzed via untargeted LC-MS-based metabolomics approach. PCA, PLS-DA, and OPLS-DA models were fitted to the resulting data as part of multivariate analysis. Differential metabolites among the three comparative root groups were identified by combining multivariate analysis with univariate analysis. These differential metabolites were further subjected to a KEGG pathway analysis^31^, which showed that some metabolic pathways were disturbed in the course of root development. Our comprehensive study provides valuable insights into the molecular basis of tuberous root development and provides critical information for assessing the quality of *R. glutinosa* roots and determining its harvest time. Our study also reveals that *R. glutinosa* root development is a complex process involving many differential metabolites that interact with many pathways and enzymes. In addition, these identified differential metabolites will provide valuable information that could be used in future comparative analyses of plant species closely related to *R. glutinosa*.

## Materials and Methods

### Plant material

The cultivar “Jinjiu (03–2)” of *R. glutinosa* was planted in farmed land in Wen County, Henan, China. Its roots from its plants at three developmental stages, elongation stage (E), expanding or thickening stage (T), and mature stage (M), were collected on May 20, August 20, and November 10, 2016, respectively. These samples were labeled elongating root (ER), expanding or thickening root (TR), and mature root (MR).

### Chemicals

All chemicals and solvents were analytical or HPLC grade. Water, methanol, acetonitrile, formic acid, and ammonium bicarbonate were purchased from CNW Technologies GmbH (Düsseldorf, Germany). The L-2-chlorophenylalanine was purchased from Shanghai Hengchuang Bio-technology Co., Ltd. (Shanghai, China).

### Sample preparation

An accurately weighed 100-mg sample was transferred to a 1.5-mL Eppendorftube, to which two small steel balls were added. A 20-μL internal standard ofmethanol and water (1/1, v/v) and 1-mL mixture of methanol and water (7/3, v/v) were added to each sample, after which allsamples were placed at −80 °C for 2 min. Samples were then ground at 60 Hzfor 2 min, put under vortexmovement for 2 min and ultrasonicated for 30 min at ambient temperature, and placed at 4 °C for 10 min. Samples were centrifuged at 14000 rpm and 4 °C for 10 min. The ensuing supernatant (500 μL) from each tube was collected using a crystal syringe, then filtered through 0.22-μmmicrofilters and transferred to LC vials. These vials were stored at −80 °C until the LC-MS analysis.

The quality control(QC) samples were prepared by mixing aliquots of all samples tocreate a pooled sample. Carnitine C2:0-d3, carnitine C8:0-d3, carnitine C10:0-d3, carnitine C16:0-d3, LPC 19:0, FFA C16:0-d3, FFA C18:0-d3, CDCA-d4, CA-d4, Trp-d5, Phe-d5, SM12:0, and choline-d4 were added to the QC samples for the retention time calibration.

### LC/MS analysis

The UHPLC system, an Ultimate 3000-Velos Pro coupled with LTQ Orbitrap MS (Thermo Fisher Scientific, Waltham, MA, USA), was used to perform and analyze the metabolic profiling in both the ESI positive and negative ionization modes. In the former mode, the separation of metabolites was conducted on a 2.1 × 100 mm ACQUITY^TM^ 1.7 μm BEH C8 column, and the mobile phase contained water with 0.1% formic acid (A) and acetonitrile (B). The linear elution gradient program was used as follows: 5% B kept for 1.0 min, linearly increased to 100% B for 24 min, and then held for 4 min, 100–5% B for 28.0 to 28.1 min, and 5%B held for 28.1 to 30 min. Each run time lasted 30 min. In the negative ionization mode, the metabolite separation was performed on 2.1 × 100 mm ACQUITY^TM^ 1.8 μm HSS T3 column, and the mobile phase contained a 6.5-mM ammonium bicarbonate water solution (C) and 6.5 mM of ammonium bicarbonate in 95% methanol and water (D). The linear elution gradient program was 5% D kept for 1.0 min, then linearly increased to 100% D for18 min, and held for 4 min, 100–5% D for 22.0 to 22.1 min, and 5% D held for 22.1 to 25.0 min. Each run timelasted 25 min.

The flow rate was set to 0.35 mL/min and the column temperature was maintained at 50 °C. The injection volume was 5 μL. Mass spectrometry detections were set as follows: a capillary temperature of 350 °C and 360° Crespectively for the positive and negative ionization modes, with a corresponding spray voltage of 3.5 kV and 3.0 kV. The mass scan had a range of 50 to 1000 m/z feature. The resolution of the MS was set to 30000. The QCs were injected at regular intervals (once every 10 samples) throughout the analytical run to provide a set of data from which repeatability could be reliably assessed.

### Data preprocessing and statistical analyses

The MS data acquired from the UHPLC-LTQ Orbitrap were analyzed in XCMS software, which produced a matrix of features with the associated retention times, accurate masses, and chromatographs^32^. The variables presentin at least 80% of either group were extracted. Those variables with a < 30% relative standard deviation (RSD) in the QC samples were kept for further multivariate data analysis, since these were considered stable enough for a prolonged UHPLC-LTQ analysis. The internal peaks were removed from the dataset. The resulting data were normalized to the total peak area of each sample with Microsoft Excel 2007 software (Microsoft, Washington, USA).

Data were imported into SIMCA (**v**14.0, Umetrics, Umea, Sweden), in which theprincipal component analysis (PCA), partial least-squares discriminant analysis (PLS-DA), and orthogonal partial least-squares discriminant analysis (OPLS-DA) were performed. The Hotelling’s T2 region, shown as an ellipse in the score plots of the models, defines the 95% confidence interval of the modeled variation. The quality of the models is described by the values of the R2X or R2Y and Q2 terms. R2X or R2Y is defined as the proportion of variance in the data explained by the models, and thus indicates a goodness of fit. Q2 is defined as the proportion of variance in the data predicted by the model, and thus indicates predictability, as calculated by a cross-validation procedure. A default seven-round cross-validation in SIMCA was performed throughout to determine the optimal number of principal components, and to avoid over-fitting the model to the data. The OPLS-DA models were also validated by a permutation analysis (with n = 200 runs).

### Identification of the differential metabolites

Differential metabolites were selected based on the combination of a statistically significant threshold of VIP (variable influence in projection) values obtained from the OPLS- DA model and the *P*-values from a two-tailed Student’s *t*-test on the normalized peak areas. We used the software, One-step Solution for Identification of Small Molecules in Metabolomics Studies—co-developed by Dalian Institute of Chemical Physics, Chinese Academy of Sciences, and the Dalian Chem Data Solution Information Technology Co., Ltd—to identify the differential metabolites. A reference material database, compiledby the Dalian Institute of Chemical Physics, Chinese Academy of Sciences, and the Dalian Chem Data Solution Information Technology Co., Ltd, along with the online databases of HMDB (http://www.hmdb.ca/spectra/ms/search) and METLIN (https://metlin.scripps.edu/) were used. The mass tolerance for the HMDB database search was set at 0.005 Da. A self-constructed LC-MS/MS identification system for metabolites servedas an effective technique for identifying the compounds.

### KEGG pathway analysis of identified differential metabolites

The identified differential metabolites were mapped onto the KEGG database using the Omics Bean Software (http://www.geneforhealth.com) for KEGG pathway analysis.

### Network analysis

The STRING database, a resource database of predicted functional associations between proteins and nucleic acids, was used for a network analysis of the top-10 KEGG pathways for the three comparative root groups. These network analyses aim to reveal the molecular interaction networks and mechanisms under pinning the top-10 KEGG pathways.

### Heatmap

The peak areas of 281 common differential metabolites among three comparative root groups were used to construct a heatmap in the R programming platform (R v3.1.1) as stated byJohansson^33^.

### Compliance with ethical standards

The conducted experiment complies with the laws of China.

## Electronic supplementary material

Supplementary Table S1

Supplementary Table S2

Supplementary Table S3

Supplementary Table S4

Supplementary Table S5

Supplementary Table S6
